# S-Glutathionylation of an Auxiliary Subunit Confers Redox Sensitivity to Kv4 Channel Inactivation

**DOI:** 10.1371/journal.pone.0093315

**Published:** 2014-03-27

**Authors:** Henry H. Jerng, Paul J. Pfaffinger

**Affiliations:** Department of Neuroscience, Baylor College of Medicine, Houston, Texas, United States of America; University of Houston, United States of America

## Abstract

Reactive oxygen species (ROS) regulate ion channels, modulate neuronal excitability, and contribute to the etiology of neurodegenerative disorders. ROS differentially suppress fast “ball-and-chain” N-type inactivation of cloned Kv1 and Kv3 potassium channels but not of Kv4 channels, likely due to a lack of reactive cysteines in Kv4 N-termini. Recently, we discovered that N-type inactivation of Kv4 channel complexes can be independently conferred by certain N-terminal variants of Kv4 auxiliary subunits (DPP6a, DPP10a). Here, we report that both DPP6a and DPP10a, like Kv subunits with redox-sensitive N-type inactivation, contain a highly conserved cysteine in their N-termini (Cys-13). To test if N-type inactivation mediated by DPP6a or DPP10a is redox sensitive, *Xenopus* oocyte recordings were performed to examine the effects of two common oxidants, tert-butyl hydroperoxide (tBHP) and diamide. Both oxidants markedly modulate DPP6a- or DPP10a-conferred N-type inactivation of Kv4 channels, slowing the overall inactivation and increasing the peak current. These functional effects are fully reversed by the reducing agent dithiothreitol (DTT) and appear to be due to a selective modulation of the N-type inactivation mediated by these auxiliary subunits. Mutation of DPP6a Cys-13 to serine eliminated the tBHP or diamide effects, confirming the importance of Cys-13 to the oxidative regulation. Biochemical studies designed to elucidate the underlying molecular mechanism show no evidence of protein-protein disulfide linkage formation following cysteine oxidation. Instead, using a biotinylated glutathione (BioGEE) reagent, we discovered that oxidation by tBHP or diamide leads to S-glutathionylation of Cys-13, suggesting that S-glutathionylation underlies the regulation of fast N-type inactivation by redox. In conclusion, our studies suggest that Kv4-based A-type current in neurons may show differential redox sensitivity depending on whether DPP6a or DPP10a is highly expressed, and that the S-glutathionylation mechanism may play a previously unappreciated role in mediating excitability changes and neuropathologies associated with ROS.

## Introduction

Many voltage-dependent potassium (Kv) channels inactivate in response to prolonged depolarization. The inactivation kinetics vary greatly among Kv channels, from slow delayed rectifier channels that barely inactivate in hundreds of milliseconds to fast A-type channels that inactivate completely within tens of milliseconds [1]. Fast inactivation is often produced by a “ball-and-chain” mechanism, where a cytoplasmic N-terminal segment enters and occludes the inner pore during channel opening, thereby terminating K^+^ conduction [2], [3]. This “N-type” inactivation can be mediated by N-terminal sequences contained on the pore-forming subunits or on certain Kv channel auxiliary subunits [2], [4], [5], [6], [7], [8], [9], [10], [11], [12], [13].

N-type inactivation in certain A-type channels has been shown to be reversibly suppressed by oxidation of specific N-terminal cysteine residues [7], [14], [15], [16], [17], [18], [19]. The oxidative regulation of Kv1.4 N-type inactivation has been hypothesized to be produced by disulfide bridge formation between a conserved cysteine 13 and some unknown cytoplasmic portion of the channel [7]. However, the importance of disulfide bridge formation for this effect remains unclear, since oxidants can generate reaction intermediates and by-products in addition to inducing disulfides that might also affect N-type inactivation. For example, the H_2_O_2_ analogue, tert-butyl hydroperoxide (tBHP), reacts with cysteine and produces sulfenic acid intermediate as well as sulfinic and sulfonic acids [20]. In addition, because the tripeptide glutathione (GSH) is present in high concentration in the cytoplasm, GSH can be crosslinked to a sulfenic acid intermediate in a process known as S-glutathionylation to produce protein-glutathione mixed disulfides [21], [22], [23]. S-glutathionylation of cysteine thiols can occur indirectly in a disulfide exchange reaction, by first generating glutathione disulfides (GSSG) followed by GSSG oxidation of reduced protein cysteine. The balance between the formation of mixed protein-glutathione disulfides verses protein-protein disulfides depends on two factors: the relative redox potentials between cysteine thiols and GSH and the relative concentrations of reactant and product species.

Previous findings have suggested that Kv4 channels, unlike Kv1.4 channels, do not produce redox-sensitive A-type K^+^ currents. The A-type currents generated in oocytes by heterologous expression of Kv4 mRNA alone or poly-A mRNA from rat thalamus are insensitive to H_2_O_2_ [Bibr pone.0093315-Serodio1] [14,15]. Moreover, in hippocampal pyramidal neurons, the somatodendritic subthreshold A-type current (I_SA_) mediated by Kv4 channels is also reportedly insensitive to oxidants [24], [25]. However, recent progress in our molecular understanding of the I_SA_ channel complex challenges this overly simplistic conclusion. In addition to Kv4 pore-forming subunits, I_SA_ channels contain notably two types of auxiliary subunits, the Kv channel-interacting proteins (KChIPs) and dipeptidyl peptidase-like proteins (DPLPs) [26], [27], [28], [29]. KChIP binding sequesters the Kv4 N-termini and effectively removes N-type inactivation mediated by Kv4 subunits [30], [31], [32], meaning that in many neurons, I_SA_ does not utilize N-type mechanisms. However, specific neuronal populations express two DPLP N-terminal variants (DPP6a and DPP10a) that can independently confer N-type inactivation on the Kv4-KChIP-DPLP channel complex [12], [13], [33], [34].

In this study we examined whether DPP6a- and DPP10a-mediated N-type inactivation of Kv4 channels is regulated by redox. We show that oxidation of a highly conserved N-terminal cysteine in DPP6a (Cys-13) dramatically slows inactivation kinetics and increases the peak current amplitude, effects that are fully reversed by dithiothreitol (DTT). Finally, oxidative regulation of N-type inactivation does not require formation of a protein-protein disulfide bridge with Cys-13; instead, S-glutathionylation of Cys-13 appears to be the key regulating event that mediates redox regulation of N-type inactivation.

## Materials and Methods

### Ethics Statement

The experimental procedures on *Xenopus laevis* frogs were conducted in accordance The National Institute of Health Guide for Care and Use of Laboratory Animals and with the approval of the Institutional Animal Care and Use Committee (IACUC) of the Baylor College of Medicine (Protocol Number: AN-752). Per approved protocol, every effort was made to minimize suffering.

### Molecular Biology

Plasmid constructs containing full-length cDNAs of Kv4.2, Kv4.2/Δ2-40, KChIP3a, DPP6a, DPP6K, and DPP10a have been described previously [12], [13]. Bovine Kv1.4 cDNA was obtained from Dr. Luis Colom (University of Texas- Brownsville, Brownsville, TX) and subcloned into the pT7T3D-Pac vector. Kv4.1 and Kv4.1 (C11xA) were generous gifts from Dr. Manuel Covarrubias (Thomas Jefferson University, Philadelphia, PA). Kv4.2/C320A, Kv4.2/C484A, Kv4.2/C529A, Kv4.2/C530A, Kv4.2/C588A, DPP6a/C13S, and Kv4.1 (C11xA)/C322 mutants were generated by first amplifying a DNA fragment containing the desired mutation by overlap extension and then subcloning back into the original construct. All mutations were verified by automated sequencing performed by the Baylor Core Sequencing Facility (Baylor College of Medicine, Houston, TX). Plasmids were linearized 3′ of the open reading frames using the appropriate restriction enzymes, and run-off cRNA transcripts were synthesized using either T3 or T7 mMessage mMachine kits (Ambion, Austin, TX).

### Heterologous Expression in Xenopus oocytes

The procedures for harvesting and maintaining oocytes have been described previously [13]. cRNAs were injected into oocytes using a Nanoinjector (Drummond). For ternary complexes, cRNAs for Kv4, KChIP, and DPLP were injected at a 1∶4∶4 molar ratio to promote complete assembly of pore-forming and auxiliary subunits. Injected oocytes were incubated in ND96 at 18°C overnight for electrophysiological recordings or 3–4 days for biochemical analysis.

### Oocyte Extract Preparation and Western Blotting

Oocyte extracts were prepared by placing 20 oocytes in 200 μl of homogenization solution (150 mM NaCl, 5 mM EDTA, 1% Triton X-100, 1X protease inhibitor (Roche), and 50 mM Tris-HCl at pH7.5). Oocyte lysis and lysate collection were performed as described previously [35]. 1x SDS-PAGE loading buffer was added either with or without 100 mM DTT (Invitrogen Life Technologies) for non-reducing or reducing conditions. SDS-PAGE and Western blotting were conducted according to Sambrook et al. [36]. The separated proteins were transferred to Immobilon membranes (Millipore, Billerica, MA) using manufacture's protocol, probed with primary antibodies, treated with appropriate HRP-conjugated secondary antibodies, and detected according to previously described methods [35]. The following primary antibodies were used for Western blotting: mouse monoclonal anti-Kv1.4 antibodies (Antibodies Incorporated; K13/31; 1∶1000 dilution) and rabbit polyclonal anti-DPP6 antibodies (A generous gift from Dr. Yoshio Misumi; 1∶2000 dilution).

### Testing for S-glutathionylation using biotinylated glutathione ethyl ester (BioGEE)

After incubation for 3 to 4 days, oocytes were first treated with [2-(Trimethylammonium) ethyl] methane thiosulfonate (MT-SET) (Affymetrix; 400 μM in ND96) for 1 min at room temperature (RT) to react with external cysteine sulfhydryls. To pre-load oocytes with BioGEE, stock BioGEE (Invitrogen Life Technologies; 2.5 mM in DMSO) was added to oocytes in ND96 produce a working BioGEE concentration of 250 μM, and the oocytes were allowed to incubate for 1 hr at RT, per published report [37]. Afterwards, oocytes were challenged with 0.5 mM of either tBHP or diamide for 15 min at RT. Then, stock NEM (Sigma-Aldrich; 0.5 M in ethanol) was added to a final concentration of 50 mM to quench all unreacted cysteines, and the oocytes were allowed to incubate for 10 min. Oocyte extracts were prepared as described above, and 100 μl of extracts were incubated with 50 μl of streptavidin agarose beads (Thermo Fisher Scientific, Rockford, IL) overnight at 4°C. On the following day, the beads were spun down (2,500×g for 2 min) and washed three times with 1 ml of homogenization solution. Then the beads were suspended in 50 μl of loading buffer with SDS and boiled for 3 min to release the proteins.

### Two-electrode Voltage Clamp Recordings

Whole-cell currents were elicited from injected oocytes and recorded to a desktop PC using the two-electrode voltage clamp technique as described previously [35]. Briefly, microelectrodes were fabricated from borosilicate glass capillaries using a micropipette puller and back-filled with 3 M KCl solutions. The WinWCP software (John Dempster, University of Strathclyde, Glasgow, UK) was used to control the voltage clamp amplifier (Oocyte Clamp, Warner Instruments, Hamden, CT). The data were digitized and low-pass filtered at various frequencies depending on the sampling rate. Tert-butyl hydroperoxide (tBHP, 70% from Acros Organics) and diamide (Sigma-Aldrich) solutions were prepared immediately before application and diluted to their nominal concentrations. Concentrations are described as “nominal” since the intracellular concentrations of these drugs cannot be determined precisely under our experimental conditions. When assayed with oocyte recordings, the dose-response curves of many compounds reportedly exhibit a rightward shift, and this effect has been proposed to be due to non-specific binding to the oocyte yolk and vitelline membranes [38]. Two (2) minutes were allowed between fluid exchanges.

### Data Acquisition and Analysis

Data for processing were analyzed using WinWCP and Origin 6.1 (Origin Lab Corp., Northampton, MA) softwares. Steady-state inactivation (SSI) was measured by introducing a 10-sec conditioning pulse at various potentials before pulsing to +50 mV to determine the amount of inactivation. The time course of recovery from inactivation was measured using two-pulse protocols, where channels initially held at −100 mV were subjected to a 25-ms-long inactivating step depolarization at +50 mV before returning to −100 mV for a variable duration, followed by a second pulse to +50 mV to determine the amount recovered. Calculations of peak conductance and measurements of SSI and recovery kinetics have been described previously [35]. For experimental averages, data are presented as mean ± standard error of the mean (SEM). Statistical significance was determined by comparing data sets using Student's two-tailed (independent) *t*-test and the significance level of p<0.05.

## Results

### DPP6a and DPP10a possess a highly conserved N-terminal cysteine, similar to subunits mediating redox-sensitive N-type inactivation

**Figure 1:**
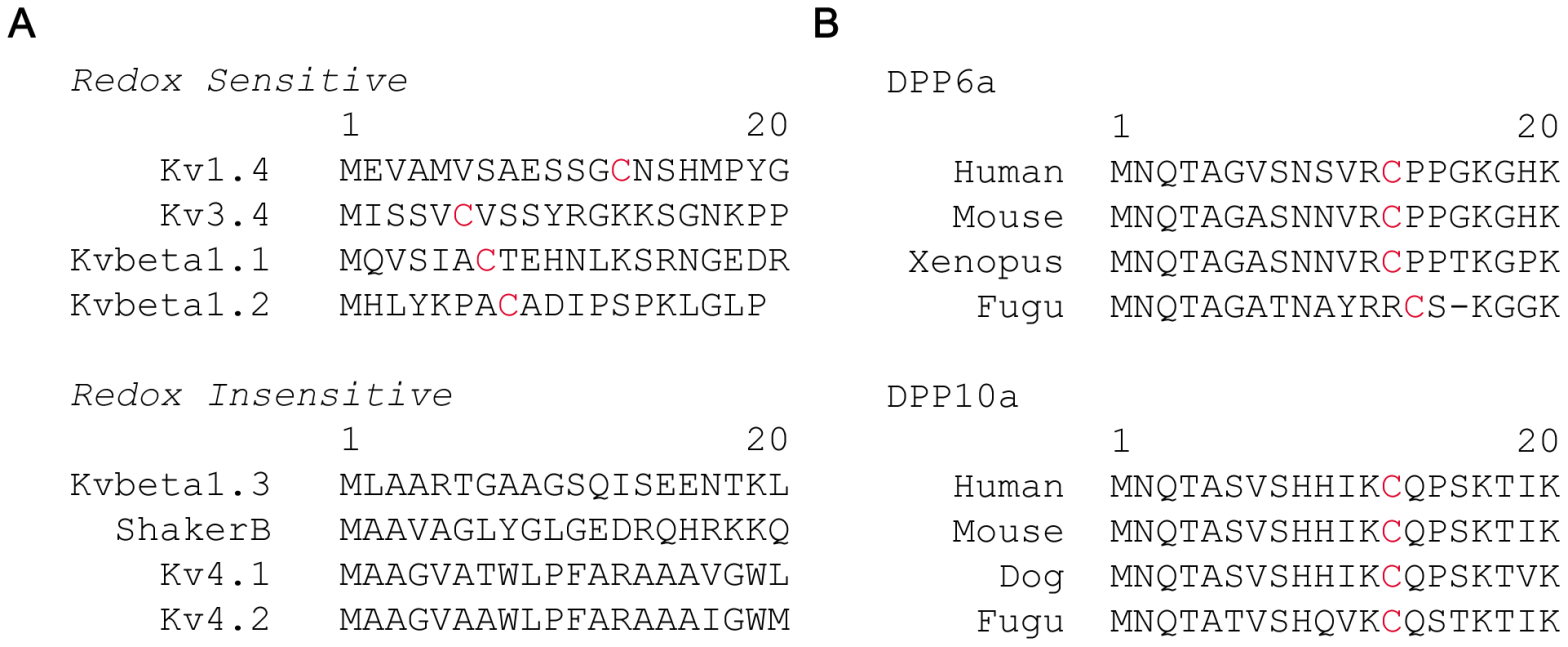
DPP6a and DPP10a N-termini possess a highly conserved cysteine (Cys-13), like other N-type inactivation domains sensitive to redox regulation. A. Alignments of the first 20 residues from the N-termini of pore-forming and auxiliary subunits known to mediate N-type inactivation. Sequences were separated into two groups based on reported redox sensitivity (see Results for reference information). B. Alignments of DPP6a and DPP10a variable N-terminal sequences (20 residues) from various species from human to fish. The unique N-terminal cysteine is highlighted in red.

N-type inactivation mediated by Kv-channel pore-forming or auxiliary subunits has been described as sensitive or insensitive to redox (Fig. 1A) [7], [14], [15], [16], [17], [19], [39]. Where redox sensitivity has been observed, this sensitivity is derived from reactive N-terminal cysteines [7], [17], [40]. To determine if N-type inactivation mediated by DPP6a and DPP10a is likely redox sensitive, we aligned their N-terminal sequences to look for conserved cysteine residues and discovered a highly conserved cysteine at position 13 (Cys-13) for both DPP6a and DPP10a (except for Fugu DPP6a, which has the cysteine at position 14) (Fig. 1B). Oxidation of a cysteine at this identical position in Kv1.4 has been shown to be responsible for slowing inactivation and increasing peak current [7], suggesting that DPP6a and DPP10a-mediated N-type inactivation may also be sensitive to redox regulation.

### Regulation of DPP6a-mediated N-type inactivation tert-butyl hydroperoxide (tBHP), an analog of the native oxidant hydrogen peroxide

**Figure 2:**
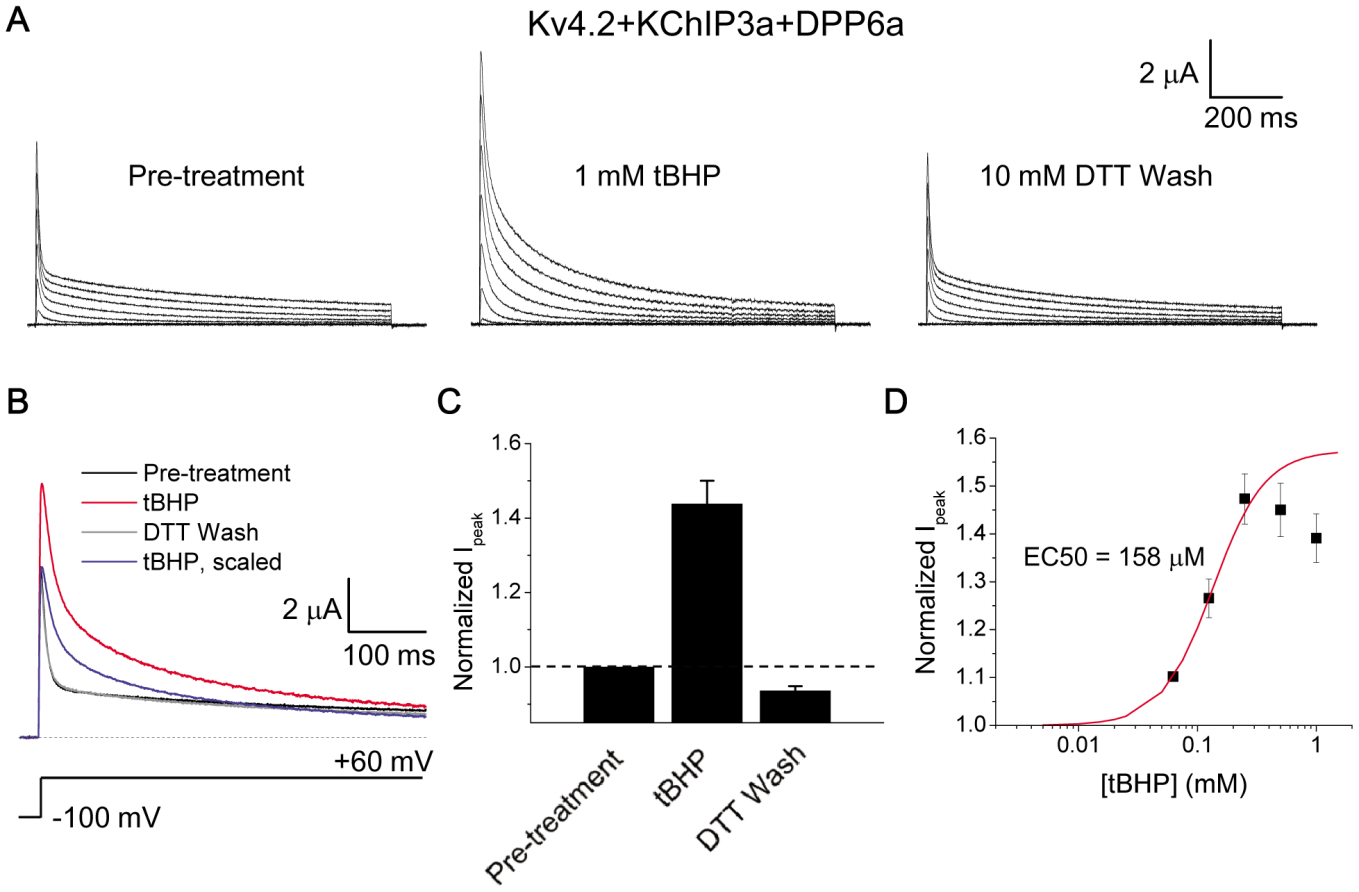
Oxidation by tBHP, a membrane-permeant analog of H_2_O_2_, reversibly increases peak current and slows DPP6a-mediated N-type inactivation. A. Representative families of whole-oocyte currents elicited before tBHP treatment, after tBHP treatment, and after subsequent reduction by DTT. Step depolarizations were made from a holding potential of −100 mV to various voltages up to +60 mV in 20 mV increments for 1 sec. B. Superimposed 500-ms-long current traces at +60 mV under the described conditions. Post-treatment trace was scaled to the pre-treatment trace to emphasize the impact on inactivation kinetics. C. Fractional changes in peak current amplitude at +60 mV after tBHP (n = 11) and DTT (n = 5) treatments. The dashed line represents no-change. D. Concentration dependence of oxidative regulation by tBHP, as measured by normalized peak current amplitude. The rising phase was fitted with a Boltzmann distribution, where the midpoint is the nominal EC50.

To test whether DPP6a- and DPP10a-mediated N-type inactivation is sensitive to redox, DPP6a was co-expressed with Kv4.2 and KChIP3a in *Xenopus* oocytes, and whole-oocyte currents were elicited by families of step depolarizations under the voltage-clamp configuration. As shown previously, although KChIP3a binds to the Kv4.2 N-terminus and eliminates the endogenous rapid N-type inactivation, the presence of DPP6a in the channel complex results in a prominent multi-phasic current decay with a dominant fast inactivating phase (τ_inact_ ∼6 ms) produced by N-type inactivation mediated by the DPP6a N-terminus (Fig. 2A, left panel) [12]. After exposure for 2 min to 1 mM tBHP, a highly membrane-permeable H_2_O_2_ analog [41], the outward currents displayed a significant slowing of inactivation kinetics and an increase in current amplitudes (Fig. 2A, middle panel). As shown in more detail at a step to +60 mV, the current decay after tBHP treatment clearly slowed markedly when compared to the pre-treatment control current, while the peak current increased dramatically by 43.8±6.1% (Figs. 2B & 2C). To test whether tBHP is acting through an oxidative mechanism, we applied the reducing agent DTT (10 mM) to tBHP-treated channels and observed a full reversal of the tBHP effects to the pre-treatment current decay kinetics and amplitude (Fig. 2A, right panel; Fig. 2B; Fig. 2C). tBHP sensitivity was evaluated by dose-response analysis, which yielded a progressive increase in effect up to approximately 250 μM (EC50 of 158±30 μM, n = 3) above which the enhancement of amplitude began to slightly decline (Fig. 2D).

**Figure 3:**
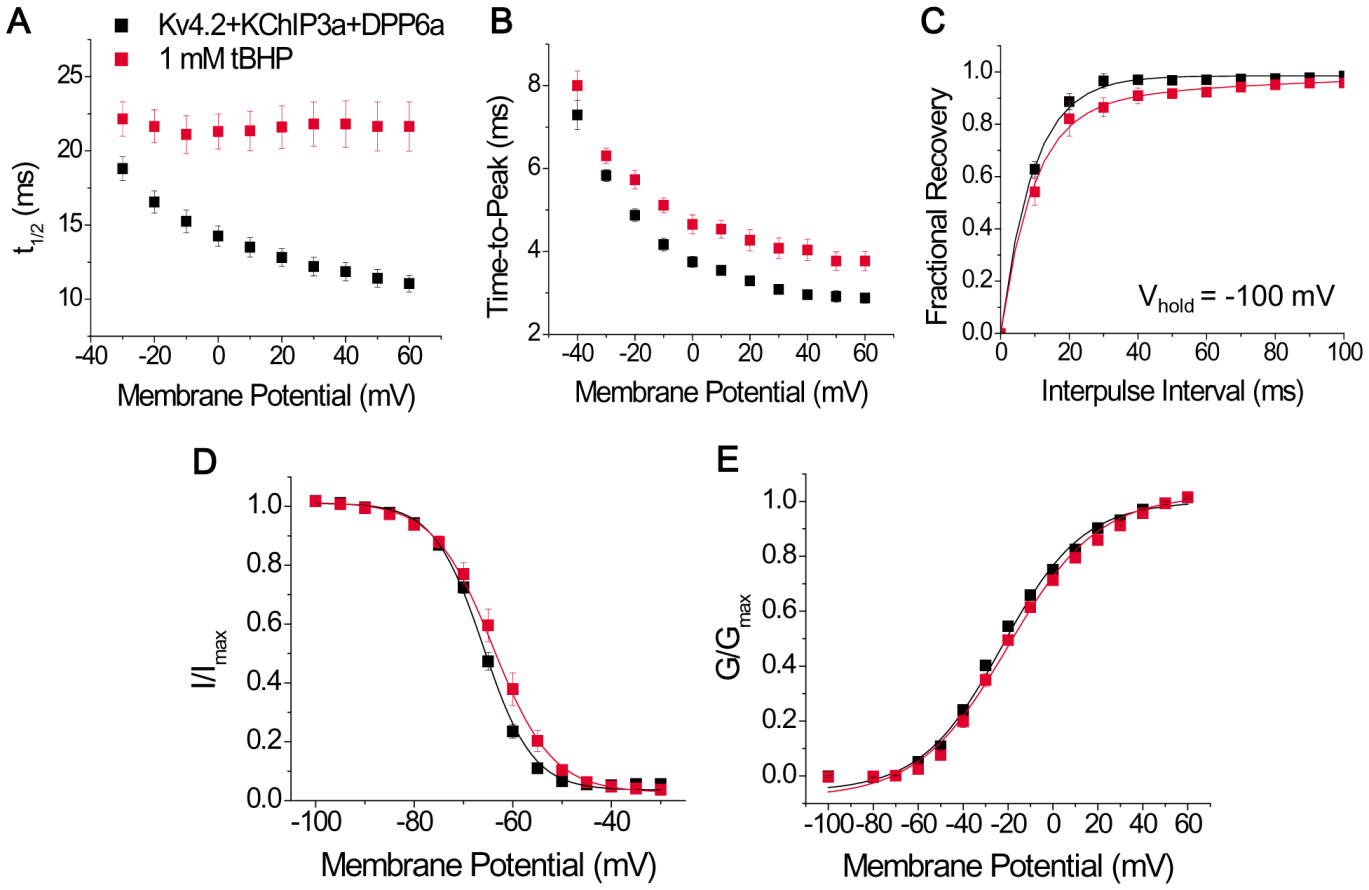
tBHP effects are mediated by slowing the inactivation time course. Biophysical properties of Kv4.2+KChIP3a+DPP6a currents were compared before (black symbols) and after (red symbols) tBHP treatment. A. Voltage dependence of the time point where half of the current is inactivated (t_1/2_). The difference between the means at +60 mV is significant at p = 0.00001. B. Voltage dependence of the time required to reach peak current. p = 0.003 for +60 mV values. C. Fractional recovery at −100 mV as a function of interpulse duration in a two-pulse recovery protocol. The curves represent single exponential fits, with time constants that are not significantly different (p = 0.178). D. Voltage dependence of steady-state inactivation. Both the midpoint shift and slope change are significant at p = 0.042 and p = 0.000094, respectively. E. Voltage dependence of relative peak conductance. No significant change was detected, with p = 0.076. In both panels D and E, the solid lines represent best fits using first-order Boltzmann functions. See Experimental Procedures for exact recording protocols.

To fully characterize the extent of tBHP's effects, various functional parameters of the expressed channels were measured, beginning with the time course of inactivation. Since N-type inactivation underlies the majority of the current decay, we avoided the unnecessary complexity of multi-exponential fittings by measuring the time required for half of the current to decay (t_1/2_). At +60 mV, the t_1/2_ values for Kv4.2+KChIP3a+DPP6a currents before and after tBHP treatment were respectively 11.05±0.56 ms (n = 10) and 21.65±1.7 ms (n = 10). As Figure 3A shows, the effects of tBHP increase as the membrane potential becomes more depolarized, consistent with tBHP modulation of N-type inactivation rather than the slow closed-state inactivation that dominates at more negative potentials. Furthermore, the slowing of inactivation does not appear to be a consequence of slowed activation since the activation kinetics, as inferred by the time to reach peak amplitude (time-to-peak), are unaffected at more negative potentials and barely slowed at positive potentials (Fig. 3B). This slightly slowing of time-to-peak at positive potentials (time-to-peak at +60 mV: control  = 2.9±0.1 ms, n = 12; +tBHP  = 3.8±0.2 ms, n = 13) is likely secondary to the N-type inactivation slowing which allows channels to continue to build up into the activated state before finally being overcome by the slow residual inactivation processes. Recovery from inactivation, as measured by a two-pulse protocol with a brief 25-ms inactivating pulse, is only slightly affected by tBHP (τ_rec_ at −100 mV: control = 9.6±1.2 ms, n = 4; +tBHP = 13.9±2.8 ms, n = 3), suggesting oxidation specifically slows the inactivation ON rate (Fig. 3C). Finally, steady-state inactivation (SSI) and conductance-voltage (g-V) relationship curves show minimal impact of tBHP, with the SSI curve exhibiting a moderate but significant ∼4 mV rightward shift and shallower slope (control: V_0.5i_ = −66.3±0.9 mV, S_i_ = 4.33±0.10 mV/e-fold, n = 9; +tBHP: V_0.5i_ = −62.5±0.9 mV, S_i_ = 5.40±0.02 mV/e-fold, n = 3) and the g-V curve showing no statistically significant change (control: V_0.5a_ = −21.0±0.8 mV, n = 9; +tBHP: V_0.5a_ = −18.0±1.6 mV, n = 4) (Fig. 3D & 3E). The moderate effect of tBHP on SSI is consistent with previous studies suggesting that this curve is primarily shaped by inactivation mechanisms that operate at more negative potentials such as closed-state inactivation [42]. Overall, these results suggest that the oxidative regulation by tBHP acts primarily on the ON rate of N-type inactivation mediated by DPP6a, and it acts by slowing inactivation kinetics without affecting channel activation or recovery from inactivation.

**Figure 4:**
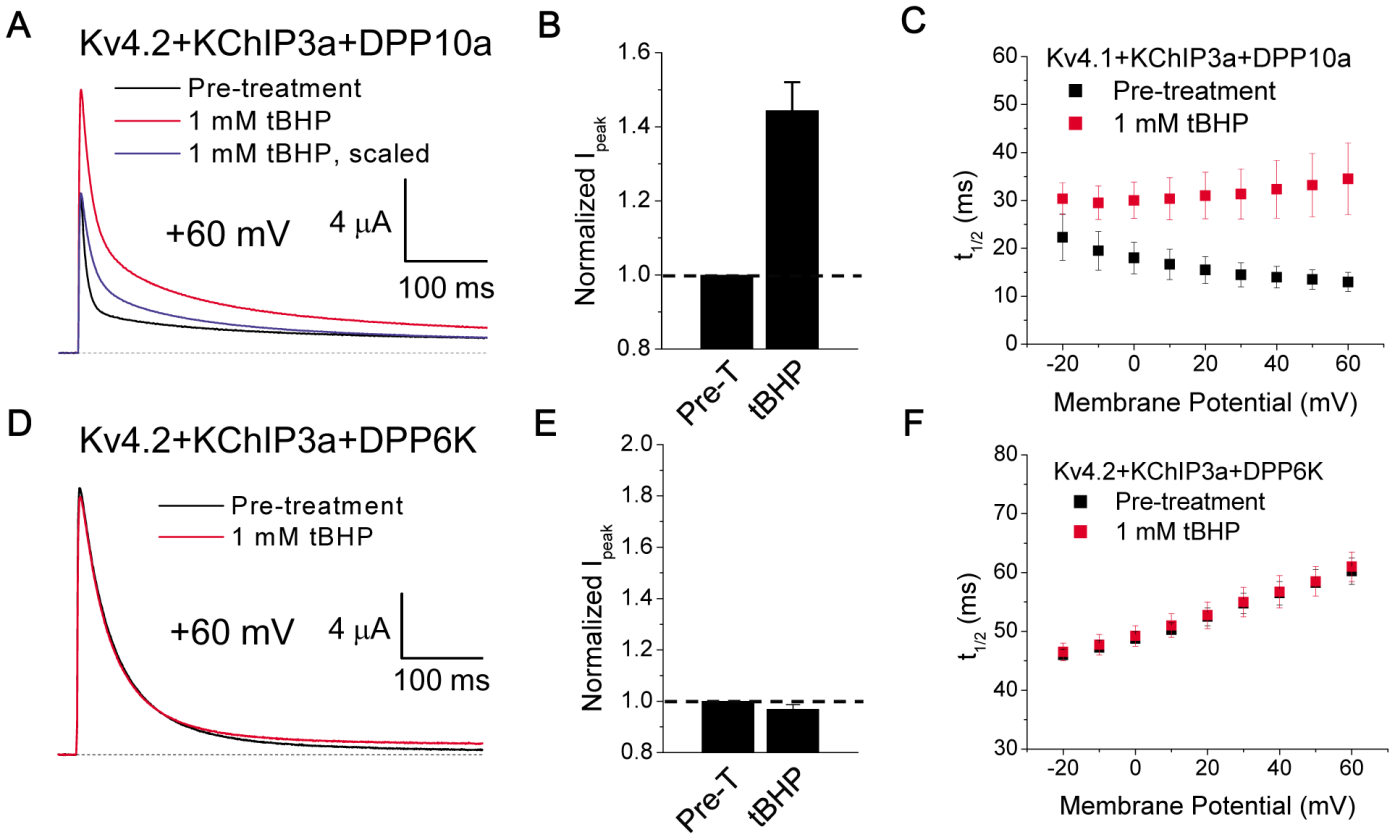
Oxidative regulation also occurs with DPP10a-mediated N-type inactivation but does not affect inactivation in the presence of DPP6K. A. Superimposed 500-ms-long Kv4.2+KChIP3a+DPP10a current traces at +60 mV before and after tBHP treatment. Post-treatment trace was scaled to pre-treatment trace to show the slowing of inactivation kinetics. B. Quantitation of increased peak current amplitude as observed in (A). The dashed line indicates no-change in amplitude. With tBHP, n = 5. Pre-T =  Pre-treatment. C. tBHP slows inactivation of Kv4.2+KChIP3a+DPP10a currents, as measured by t_1/2_, throughout the voltage range examined. At +60 mV, the difference between means is significant at p = 0.05. D. Ternary complex containing DPP6K is insensitive to oxidative regulation by tBHP. Current traces at +60 mV before and after tBHP treatment were overlapped. E. tBHP has no effect on peak current amplitude in the presence of DPP6K. With tBHP, n = 4. F. tBHP has no effect on inactivation of ternary channel complex with DPP6K.

When DPP6a was replaced by DPP10a, a similar sensitivity to tBHP was found, suggesting a common regulatory mechanism. For channels expressed with DPP10a, treatment with 1 mM tBHP increases peak current by 44.4±7.6% (Fig. 4A & 4B) and dramatically slows overall inactivation (t_1/2_ at +60 mV: control  = 13±2 ms, n = 3; +tBHP = 34.5±7.5 ms, n = 3) (Fig. 4A & 4C). The EC50 of tBHP effects was 115±12 μM (n = 4), which is not statistically significantly different from channels expressed with DPP6a (p = 0.19), and the slowing of inactivation becomes more pronounced with increasing depolarization as predicted. To determine if tBHP can modulate Kv4.2 slower inactivation processes by oxidizing amino acid residues not involved in DPP6a- or DPP10a-mediated N-type inactivation, we tested tBHP on ternary complex channels incorporating the DPP6K variant that does not produce N-type inactivation [13]. Treatment of these channels with 1 mM tBHP has no effect on peak current or inactivation kinetics (Fig. 4D–4F); thus, intrinsic slow inactivation processes of Kv4.2 channels, such as vestigial P/C-type or closed-state inactivation, are not modulated by tBHP. Overall, these results indicates that indeed oxidation by tBHP selectively disrupts the N-type inactivation of Kv4 channels produced by DPP6a or DPP10a.

### Cys-13 is the target of oxidative regulation

**Figure 5:**
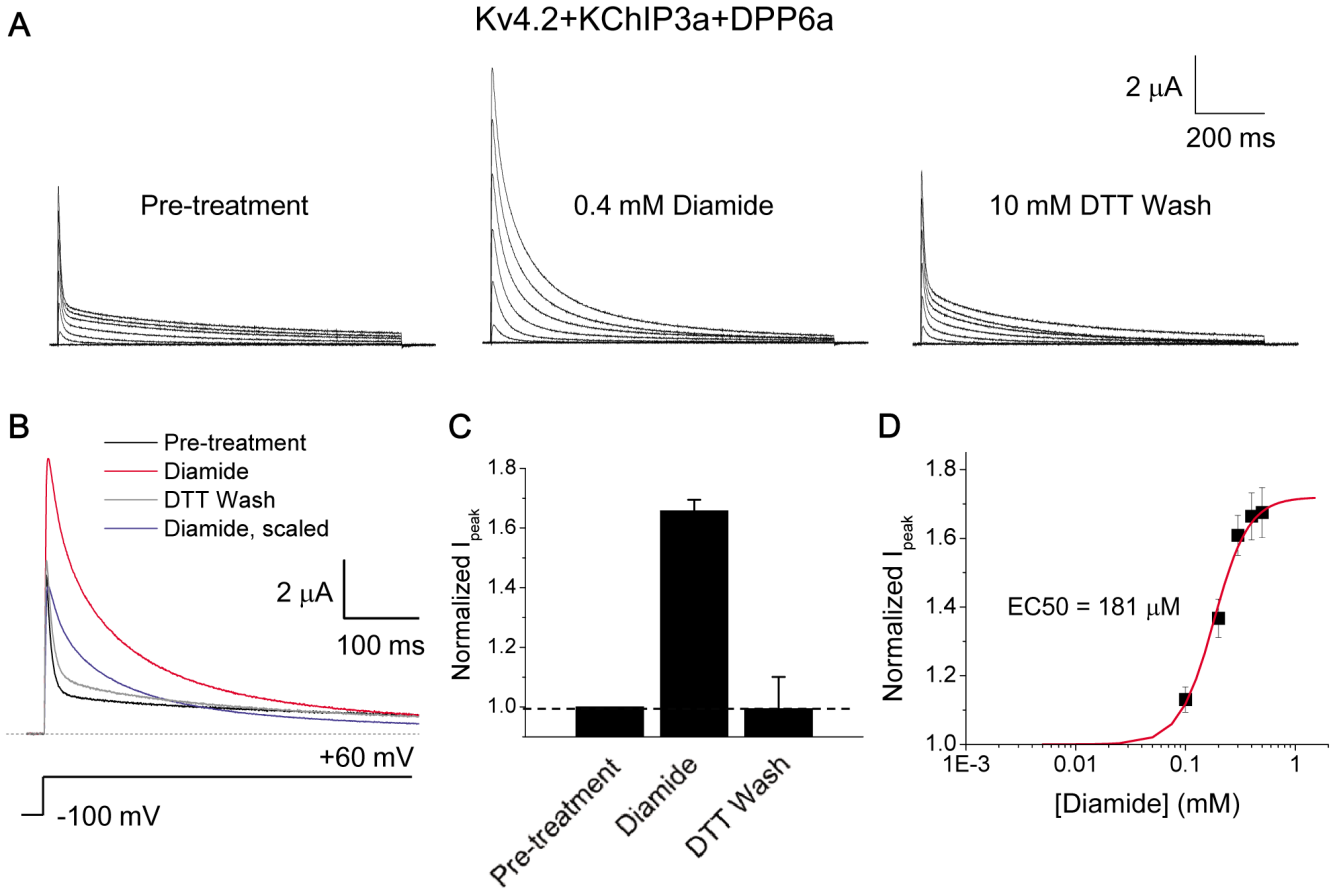
Diamide specifically react with cysteines and produces the same effects as tBHP with similar concentration dependence. A. Outward currents expressed by Kv4.2+KChIP3a+DPP6a channels in response to 1-sec step depolarizations from −100 mV holding potential to +60 mV in 20 mV increments. B. Overlapped 500-ms-long current traces at +60 mV before and after diamide exposure. Post-treatment trace was also scaled to that of pre-treatment trace to illustrate the dramatic slowing of inactivation. C. Relative change in peak current amplitude at +60 mV in response to oxidation by diamide and reduction by DTT. With diamide, n = 6; DTT washout, n = 3. D. Concentration dependence of oxidative regulation by diamide, as measured by normalized peak current amplitude. The fitted trace is the best fit using a first-order Boltzmann function.

Like H_2_O_2_, tBHP can oxidize methionines as well as cysteines [41], [43]. To narrow the target of oxidative regulation down to just cysteines, we exposed Kv4.2+KChIP3a+DPP6a channels to diamide, a thiol-specific oxidizing agent [44]. Treatment of the channels with 0.4 mM diamide rapidly increased peak current and slowed inactivation, modifications that are reversed by 10 mM DTT (Fig. 5A). Overlapped and scaled traces at +60 mV show the dramatic effects of diamide oxidation, with the peak current increased by 66±4% and t_1/2_ increased significantly by 5.6-fold (t_1/2_ at +60 mV: control  = 10.2±0.73 ms, n = 3; +diamide  = 56.7±1.9 ms, n = 3) (Fig. 5B & 5C). The diamide dose-response curve indicates that the EC50 is 182±11 μM (n = 3), which is not statistically different from the EC50 for tBHP (p = 0.50) (Fig. 5D). One notable difference when compared to tBHP is that diamide concentrations higher than 250 μM do not produce a reduction in peak current, suggesting that methionine oxidation may be responsible for the peak current decline at higher tBHP concentrations (see Discussion).

**Figure 6:**
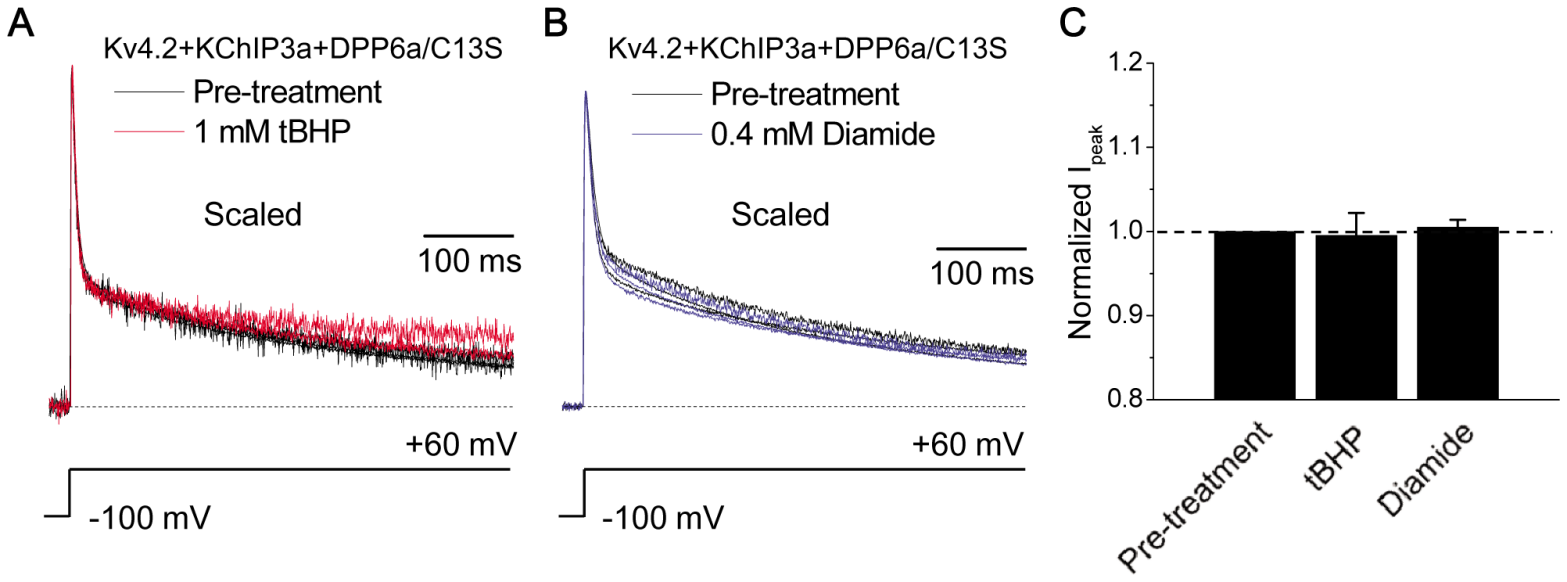
Cys-13 on DPP6a is the target of both tBHP- and diamide-induced oxidation. A. Kv4.2+KChIP3a+DPP6a/C13S currents in response to 500-ms-long depolarization at +60 mV before and after 1 mM tBHP treatment. Multiple traces are overlapped to show that the current is no longer sensitive to tBHP. B. Response of 500-ms-long Kv4.2+KChIP3a+DPP6a/C13S current at +60 mV to 0.4 mM diamide. Again, overlapped multiple traces show that the mutation C13S renders DPP6a insensitive to diamide. C. Quantitative measurements of change in peak current before and after oxidant treatment. With tBHP, n = 3; with diamide, n = 7.

To directly test the importance of Cys-13 for tBHP and diamide oxidative effects, we mutated Cys-13 on DPP6a to a serine (DPP6a/C13S). The C13S mutation does not significantly affect N-type inactivation produced by DPP6a (t_1/2_: Kv4.2+KChIP3a+DPP6a = 10.2±0.73 ms, n = 3; Kv4.2+KChIP3a+DPP6a/C13S = 10.8±0.4 ms, n = 5) (p = 0.17), suggesting that Cys-13 is not critical for N-type inactivation or co-assembly with the channel complex. However, the mutation eliminated the oxidative regulation of N-type inactivation kinetics and peak current amplitude produced by tBHP or diamide (Fig. 6A–6C). Compared to control, the t_1/2_ values were not significantly different after tBHP (p = 0.16986) or diamide (p = 0.38458) treatment. As would be expected, tBHP did not produce any significant effects on other measured biophysical properties of Kv4.2+KChIP3a+DPP6a/C13S channels, including recovery kinetics (τ at −100 mV: control  = 8.1±0.3 ms, n = 5; +tBHP = 6.5±0.3 ms, n = 3), g-V relations (control: V_0.5a_ = −23.6±4.2 mV and S_a_ = 20.7±1.7 mV/e-fold, n = 3; +tBHP: V_0.5a_ = −21.0±1.8 mV and S_a_ = 20.6±1.7 mV/e-fold, n = 3), and steady-state inactivation (control: V_0.5i_ = −65.4±1.0 mV and S_i_ = 4.35±0.11 mV/e-fold; +tBHP: V_0.5i_ = −66.4±0.8 mV and S_i_ = 4.71±0.05 mV/e-fold, n = 3). We conclude that tBHP and diamide act by oxidizing Cys-13 to produce the observed slowing of N-type inactivation and increased current amplitude.

### Regulation of inactivation by oxidation of DPP6a Cys-13 does not require disulfide bridge formation

**Figure 7:**
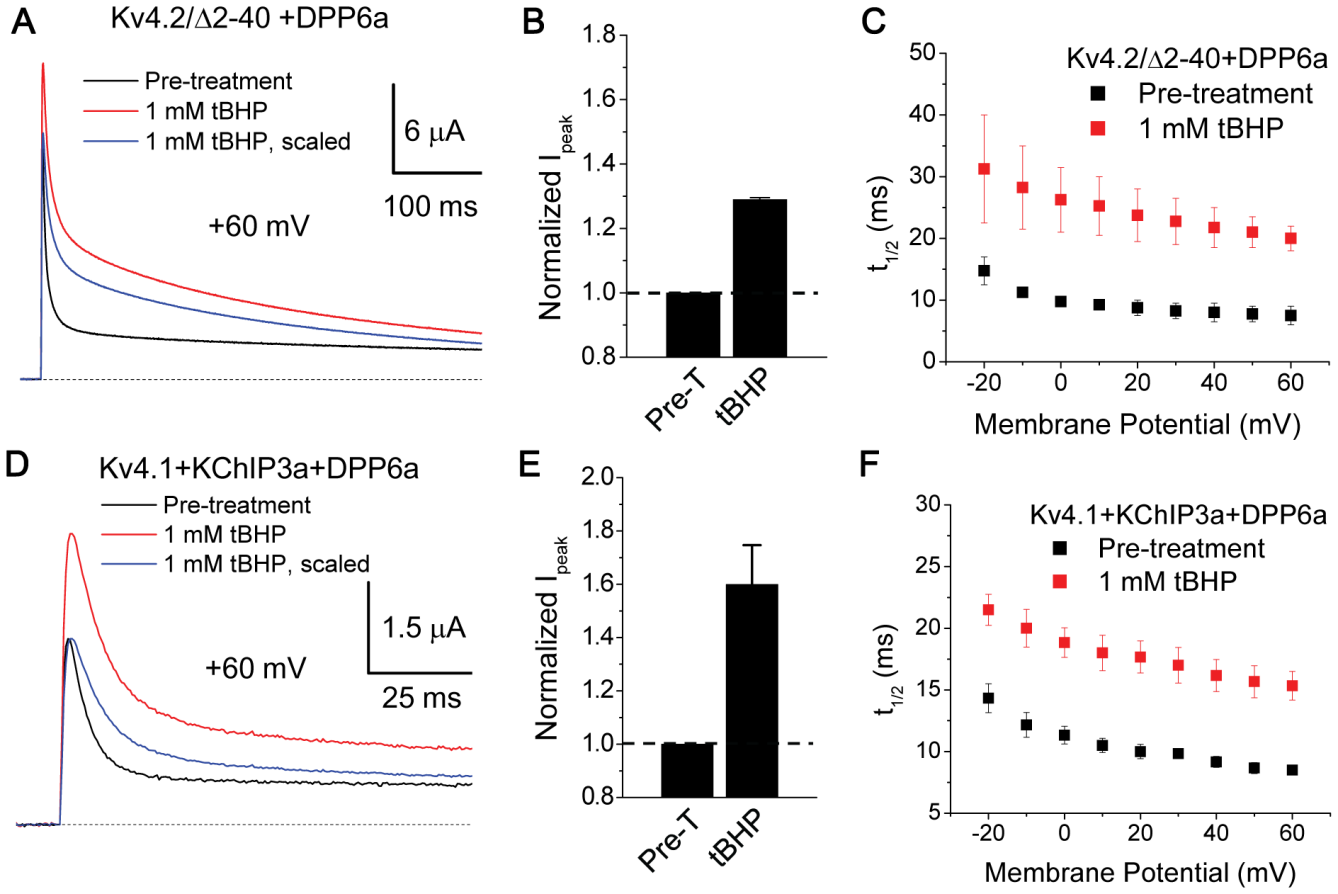
Oxidative regulation does not depend on KChIP3a or specific Kv4 subunit. A. Superimposed 500-ms-long outward current expressed by Kv4.2/Δ2-40+DPP6a channels in the presence and absence of 1 mM tBHP. B. tBHP increases peak current amplitude at +60 mV, as seen in (A). With tBHP, n = 3. C. t_1/2_ measurements, showing that tBHP slows inactivation throughout the voltage ranged tested. At +60 mV, difference is statistically significant (p = 0.038). D. Superimposed 500-ms-long Kv4.1+KChIP3a+DPP6a current traces at +60 mV before and after tBHP treatment. Substitution of Kv4.1 for Kv4.2 does not alter the ability of tBHP to increase peak current and slow inactivation. E. Quantitation of increased peak current amplitude as observed in (D). With tBHP, n = 4. Dashed line represents no increase in current. F. tBHP slows inactivation of Kv4.1+KChIP3a+DPP6a currents over the voltage ranged tested. At +60 mV, the difference is statistically significant, with P = 0.005. Pre-T =  Pre-treatment.

Oxidation of DPP6a Cys-13 can lead to a number of possible products, primarily sulfinic/sulfonic acids, intra-protein disulfides, inter-protein disulfides, and S-glutathionylation. Since the reactions involving diamide do not generate cysteine sulfenic acid [45], [46] and functional effects of tBHP and diamide are readily reversed by DTT in our experiments (Fig. 2A & 5A), it is unlikely that oxidative regulation occurs by the irreversible formation of cysteine sulfinic or sulfonic acids. Intra-protein disulfide bond formation is also unlikely, since DPP6a and DPP10a only have a single cysteine in their exposed cytoplasmic N-terminal domains (Fig. 1B). However, inter-protein disulfides are possible between Cys-13 and cysteines on members of the I_SA_ channel complex or other closely associated proteins. Since KChIPs are important I_SA_ channel components that suppress endogenous Kv4.2 N-type inactivation, we first sought to determine if KChIP is required for the oxidative regulation of DPP6a-mediated N-type inactivation. To perform experiments without KChIPs, DPP6a was co-expressed with a Kv4.2 mutant with an N-terminal truncation (Kv4.2/Δ2-40) that eliminates the endogenous Kv4.2 N-type inactivation and allows us to focus on the DPP6a-mediated N-type inactivation. As Figure 7A shows, even in the absence of KChIP3a, oxidation of DPP6a by tBHP increased peak current amplitude and slowed inactivation. Our measurements show that with 1 mM tBHP peak current at +60 mV increased by 28.9±1% and the t_1/2_ values also increased dramatically throughout the voltage ranged tested (t_1/2_ at +60 mV: control  = 7.5±1.5 ms, n = 3; +tBHP = 20±2 ms, n = 3)(Fig. 7B & 7C). The results clearly suggest that DPP6a Cys-13 does not need to form a disulfide bridge with KChIP3a to regulate N-type inactivation.

Next, to test if the regulation requires a specific reactive cysteine from the Kv4 subunit, we substituted Kv4.2 with Kv4.1 in the ternary complex and challenged the channels with tBHP. Of the 16 cysteines found in the Kv4.2 sequence, three out of the 11 cysteines on the cytoplasmic domains (Cys-503, Cys-563, Cys-583) are not conserved in Kv4.1; therefore, the Kv4.1-for-Kv4.2 substitution should eliminate the oxidative regulation of DPP6a if these cysteines were involved. However, substituting Kv4.1 for Kv4.2 in the Kv4-KChIP3a-DPP6a ternary complex does not affect the ability of tBHP to increase peak current and slow inactivation (Fig. 7D). At +60 mV, the peak current increased by 60±15% and the t_1/2_ value were significantly slowed (t_1/2_ at +60 mV: control  = 8.5±0.3 ms, n = 3; +tBHP = 15.3±1.2 ms, n = 3) (Fig. 7E & 7F).

**Figure 8:**
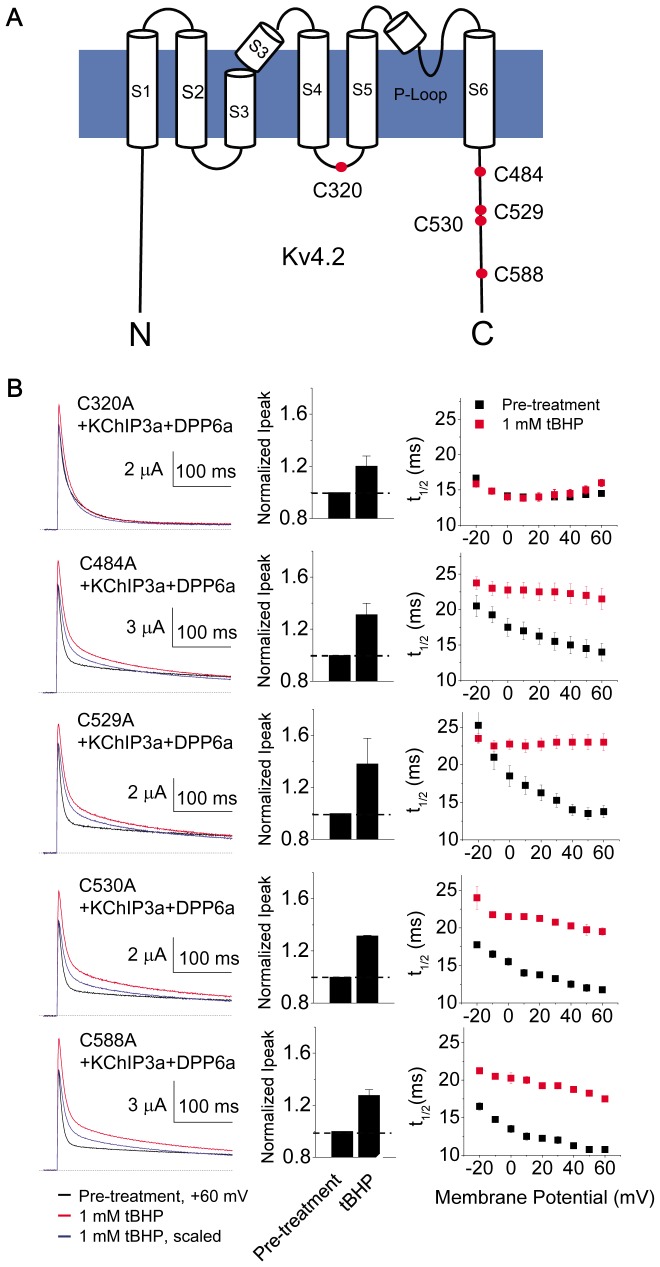
Cysteine-to-alanine mutations of cysteine residues common to Kv4.2 and Kv4.1. A. Cartoon illustration of the locations of the Kv4.2 intracellular cysteines conserved between Kv4.1 and Kv4.2 (red dots). B. Outward currents expressed by channels formed by cysteine-to-alanine mutants, KChIP3a, and DPP6a at +60 mV before and after exposure to 1 mM tBHP. (left panels) Representative overlapped traces before and after scaling. (middle panel) Quantitation of changes in current amplitude. Data for mutant channel complexes with C320A, C484A, C529A, C530A, and C588A in tBHP were collected in triplicates (n = 3). (right panel) Measurement of t_1/2_ at indicated voltages. S1–S6  =  transmembrane segments 1 through 6. N = N-terminus. C = C-terminus.

Of the 13 cysteines common between Kv4.1 and Kv4.2, three are located in the T1 domain (Cys-111, Cys-132, Cys-133) and are involved in Zn^2+^ coordination, two are located extracellularly in the S1–S2 linker (Cys-209, Cys-221), and three other cysteines are found in transmembrane segments (Cys-231, Cys-236, Cys-290) [47]. Because of their locations, these cysteines are unlikely to be a disulfide bridge partners for DPP6a Cys-13. Therefore, we focused our attention on the remaining conserved cytoplasmic cysteines: Cys-320, located in the S4–S5 linker, and four other cysteines (Cys-484, Cys-529, Cys-530, Cys-588) that are located in the cytoplasmic C-terminus (Fig. 8A). Cys-320, Cys-484, Cys-529, Cys-530, and Cys-588 were individually mutated to alanines (C320A, C484A, C529A, C530A, C588A), and the mutants were co-expressed with KChIP3a and Kv4.2 in oocytes for electrophysiological study. The results show that elimination of cysteines at positions 484, 529, 530, and 588 does not prevent tBHP-induced increases in peak current amplitude or slowing of inactivation (Fig. 8B). Unlike the other Cys-to-Ala mutations, the C320A mutation produced mixed results. This mutation caused a loss of oxidative regulation of inactivation (t_1/2_ at +60 mV: control  = 14.5±0.3 ms, n = 3; +tBHP = 16±0.5 ms, n = 3, p = 0.06) and peak current amplitude (p = 0.49), consistent with a possible role as a disulfide partner for Cys-13 (Fig. 8B). However, the C320A mutation itself markedly altered both the shape of the current decay and the voltage dependence of inactivation (Fig. 8B), indicating that C320A mutation produces other gating effects that may be masking oxidative effects on the DPP6a-mediated N-type inactivation.

**Figure 9:**
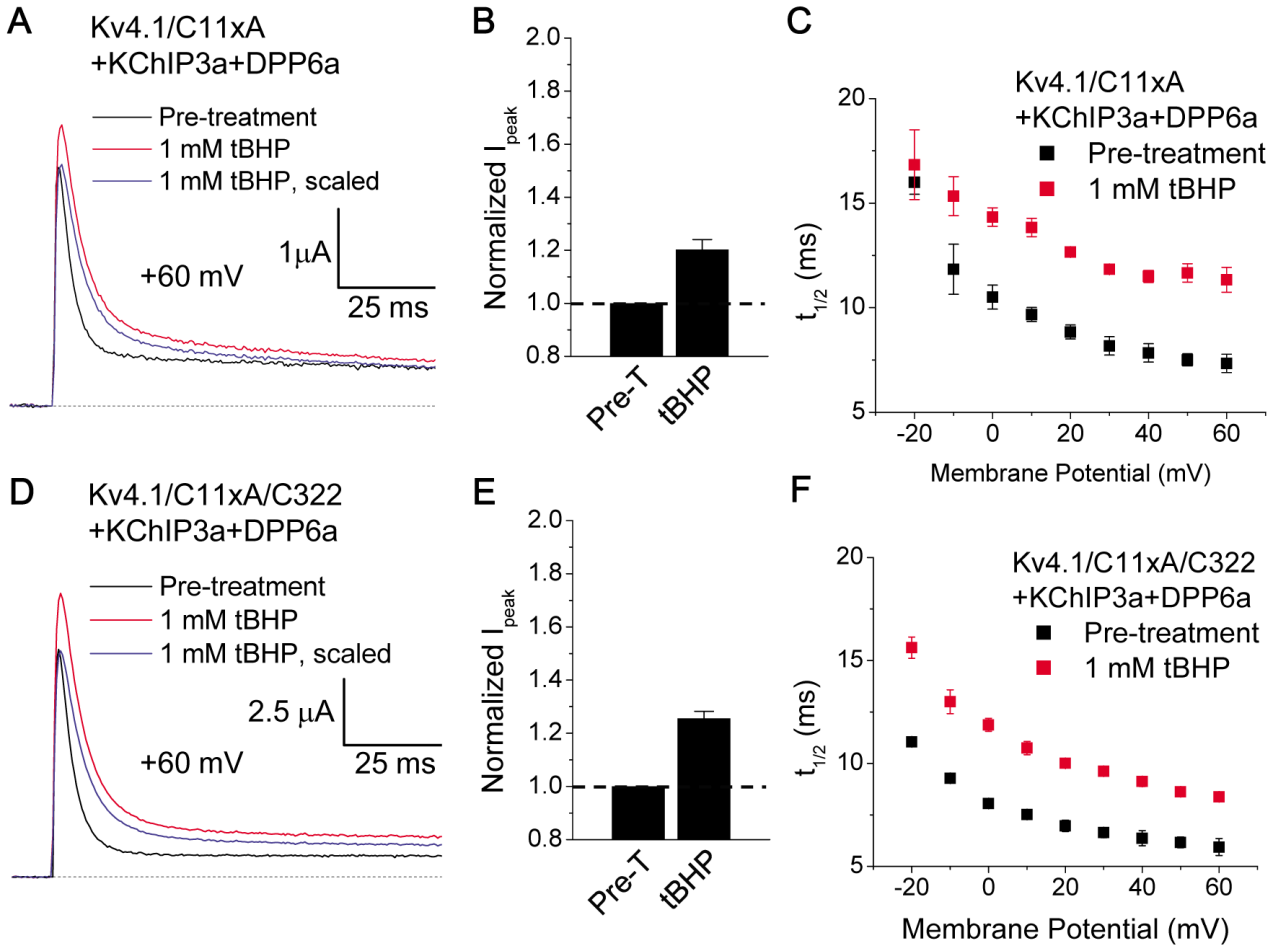
Elimination of all Kv4.1 internal cysteines not essential to expression and re-introduction of Cys-322 do not affect oxidative regulation by tBHP. A. Superimposed traces at +60 mV of Kv4.1/C11xA+KChIP3a+DPP6a channels before and after tBHP treatment. B. Increased peak amplitude produced by tBHP observed in (A). With tBHP, n = 3. C. Slowing of inactivation kinetics by tBHP indicated in (A), measured as t_1/2_. At +60 mV, the differences in the mean values are significant (p = 0.006). D. Superimposed traces at +60 mV of Kv4.1/C11xA/C322+KChIP3a+DPP6a channels before and after 1 mM tBHP. E. tBHP increases peak current amplitude at +60 mV. With tBHP, n = 4. F. tBHP slows inactivation of Kv4.1/C11xA/C322+KChIP3a+DPP6a. P = 0.002 for differences at +60 mV.

To further investigate the importance of the cysteine in the S4–S5 linker, we tested Kv4.1/C11xA, a mutant with all intracellular cysteines mutated to alanines, except the three surrounding the T1 domain zinc site (Cys-110, Cys-131, Cys-132) [47]. The T1 domain cysteines were retained because their elimination affects channel formation. The channel complex resulting from the co-expression of Kv4.1/C11xA, KChIP3a, and DPP6a exhibited multi-exponential decay and inactivated more rapidly than a channel complex with wild-type Kv4.1, with t_1/2_ at +60 mV decreasing from ∼10 ms to ∼7 ms (Compare Fig. 7F to Fig. 9C). This acceleration of inactivation has been previously reported with Kv4.1/C11xA+KChIP1+DPP6S channels [47]. Treatment of Kv4.1/C11xA+KChIP3a+DPP6a channels with 1 mM tBHP both increased peak current by 20.2±3.7% and significantly increased t_1/2_ at +60 mV from 7.3±0.4 ms (n = 3) to 11.3±0.6 ms (n = 3)(Fig. 9A–9C). The result suggests that these 11 cysteine residues on Kv4.1 channels, including Cys-322 (equivalent of Kv4.2 Cys-320), are not important for oxidative modulation. In contrast to the Kv4.2/C320A channel complex, the Kv4.1/C11xA channel complex inactivated more rapidly with increased depolarization (compare Fig. 9C with Fig. 8B). However, similar to the cysteine mutations in Kv4.2 channels, these mutations in Kv4.1 channel also resulted in a significant decrease in the magnitude of peak current increase resulting from tBHP treatment (compare Fig. 7E with 9B). Finally, Cys-322 (equivalent to Kv4.2 C320) was restored on the Kv4.1/C11xA subunits, making Kv4.1/C11xA/C322. Currents expressed by Kv4.1/C11xA/C322+KChIP3a+DPP6a channels show responses to tBHP that are indistinguishable from Kv4.1/C11xA+KChIP3a+DPP6a (Fig. 9D–9F). In response to tBHP, the peak current increased by 25.5±2.8% and t_1/2_ at +60 mV increased from 5.9±0.4 ms (n = 4) to 8.4±0.2 ms (n = 4). In summary, Kv4.1 Cys-322 is not required for oxidative regulation mediated by DPP6a Cys-13, suggesting that indeed the effects observed with Kv4.2/C320A are the results of other gating changes associated with the C320A mutation. The direct importance of Kv4.2 Cys-320 in the redox regulation mediated by DPP6a Cys-13 is unclear due to the mutation introducing an acceleration of closed state inactivation and masking the oxidative effects (see Discussion).

**Figure 10:**
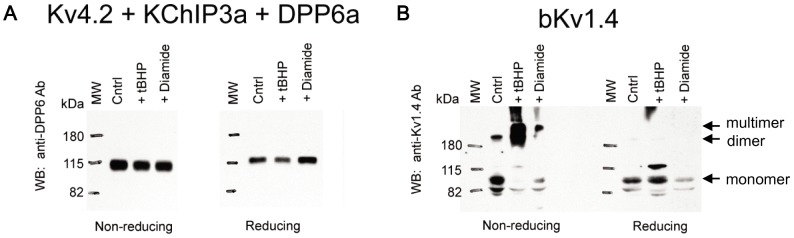
tBHP and diamide do not induce DPP6a gel shift, inconsistent with inter-molecular disulfide bridge. Protein extracts were prepared from oocytes injected with Kv4.2, KChIP3a, and DPP6a cRNAs or with bovine Kv1.4 (bKv1.4) cRNA. Proteins from extracts were separated by SDS-PAGE under reducing and non-reducing conditions, transferred onto Immobilon membranes, and probed with the indicated antibodies. A. DPP6 proteins detected from Kv4.2+KChIP3a+DPP6a protein extracts with or without oxidant treatment using anti-DPP6 antibody. B. bKv1.4 proteins detected from bKv1.4 protein extracts with and without oxidant treatment using anti-Kv1.4 antibody. Results show that DPP6a exhibits no change in migration through SDS-PAGE under non-reducing conditions, while bKv1.4 does. Cntrl  =  Control.

To directly determine if a disulfide linkage to DPP6a Cys-13 is important for oxidative regulation, we next sought to biochemically test if oxidation of Cys-13 forms a cysteine disulfide between DPP6a subunits and any other intracellular proteins. Formation of such an inter-protein disulfide should produce a shift in the migration of DPP6a on non-reducing SDS-PAGE gels. Therefore, oocytes expressing Kv4.2, KChIP3a, and DPP6a were incubated in ND96 (Cntrl), ND96 with 1 mM tBHP, or ND96 with 1 mM diamide for 15 min. Immediately after treatment, lysates were prepared, separated out on SDS-PAGE gels under either reducing or non-reducing condition, and Western blotted. Probing for DPP6 proteins shows that under non-reducing condition, DPP6 migration is not altered by oxidation by tBHP or diamide (Fig. 10A), suggesting that Cys-13 does not form a disulfide bridge to another protein, including Kv4 or KChIP3a, or another DPP6a subunit. Observed in multiple runs, DPP6 migrates slightly slower under reducing condition compared to non-reducing condition (Fig. 10A); however, this slight migration difference is not affected by oxidants and may be caused by cysteine disulfide bonds that are normally present in the large extracellular domain of DPP6 [48]. As a positive experimental control to validate the oxidizing and reducing conditions that we used in these experiments, extracts from oocytes expressing bovine Kv1.4 (bKv1.4) were prepared and processed the same way (Fig. 10B). When gels are run under reducing conditions, bKv1.4 migrates similarly with or without tBHP or diamide treatments, showing a strong monomer band of ∼98 kDa and a weaker band of ∼82 kDa (possibly incompletely reduced monomer proteins). However, when the gel was run under non-reducing conditions, an additional high-molecular-weight band greater than 180 kDa is apparent, which most likely represent dimers (Fig. 10B). After exposure to tBHP and diamide, most of the 98 kDa band disappears and is replaced by higher molecular weight forms as expected for inter-subunit cysteine disulfide linkages that generate dimers and multimers (Fig. 10B, arrows). Overall, our biochemical results show that oxidation by tBHP and diamide at experimental concentrations does not result in the formation of inter-molecular disulfides with DPP6a.

### Oxidation of DPP6a Cys-13 results in S-glutathionylation

**Figure 11:**
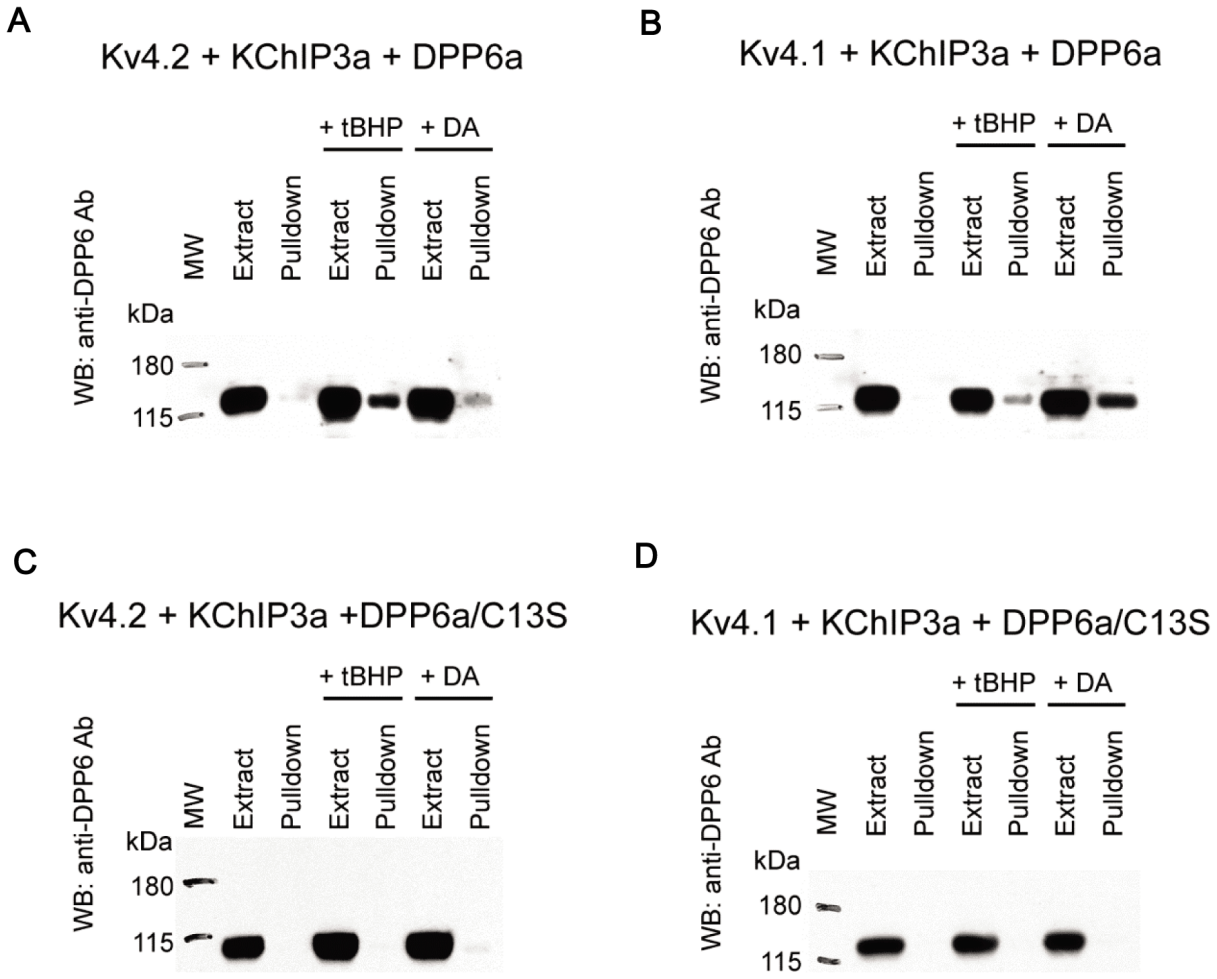
tBHP and diamide both induce S-glutathionylation of DPP6a in the Kv4 channel complex. A. Western blot loaded with Kv4.2+KChIP3a+DPP6a extract and the pulldown proteins under untreated control, tBHP, and diamide (DA) conditions. B. Western blot loaded with Kv4.1+KChIP3a+DPP6a extract and the pulldown proteins under untreated control, tBHP, and diamide conditions. C. Western blot with Kv4.2+KChIP3a+DPP6a/C13S extract and pulldown proteins under untreated control and oxidized conditions. D. Western blot with Kv4.1+KChIP3a+DPP6a/C13S extract and pulldown proteins under untreated control and oxidized conditions. In all panels, Anti-DPP6 antibody was used to detect DPP6.

tBHP and diamide have both been documented to cause extensive conversion of intracellular GSH to GSSG by oxidation, increase GSSG/GSH ratio, and enhancing the formation of glutathione-protein mixed disulfides [49], [50], [51], [52], [53]. Thus, we tested whether the oxidative regulation of DPP6a-conferred N-type inactivation by tBHP and diamide occurs by S-glutathionylation of Cys-13. Oocytes expressing channel complexes composed of Kv4.2, KChIP3a, and DPP6a were treated with the membrane-impermeable cysteine-specific MTSET reagent to block extracellular cysteines, preloaded with 250 μM BioGEE, and then oxidized by 1 mM tBHP or 1 mM diamide. To prevent further reactions, the oocytes were quenched with the cysteine-reactive NEM prior to processing for streptavidin pulldown. As Figure 11A shows, DPP6a protein is not pulled down by streptavidin beads in the absence of oxidation by tBHP or diamide. However, after oxidation by tBHP or diamide, a significant amount of DPP6a is pulled down, indicating that DPP6a is glutathionylated under the experimental conditions. The same experiment was also performed with Kv4.1 substituting for Kv4.2, to check if the DPP6a is glutathionylated in the presence of Kv4.1. Our results show that again DPP6a is not pulled down by streptavidin beads in the absence of oxidants; however, after incubation with either tBHP or diamide, DPP6a is pulled down, indicating that the oxidants induced S-glutathionylation of DPP6a (Fig. 11B). Finally, to test whether Cys-13 is the residue glutathionylated as a result of tBHP or diamide exposure, DPP6a/C13S mutant was co-expressed with KChIP3a and either Kv4.2, or Kv4.1. As Figure 11C and 11D show, the C13S mutation prevented the pull down of DPP6a, consistent with Cys-13 being glutathionylated by oxidant exposure.

## Discussion

In conclusion, oxidative regulation of DPP6a- and DPP10a-conferred N-type inactivation of Kv4 channels is likely being mediated by S-glutathionylation of Cys-13 instead of intra- or inter-protein disulfide linkage formation. The addition of glutathione to Cys-13 results in a marked slowing of N-type inactivation and a concomitant increase in peak current, with insignificant effects on recovery from inactivation and moderate effects on other channel properties. Regulation of N-type inactivation by S-glutathionylation represents a novel molecular mechanism by which Kv channels can respond to changes in the oxidative state of the cell.

### DPP6a and DPP10a mediate Shaker N-type inactivation sensitive to oxidation

Using both *Xenopus* oocyte and culture cell lines, we previously determined that the N-terminal domain of DPP10a and DPP6a functions as a fast inactivation gate with characteristics similar to that of Shaker N-type inactivation [12]. Here we further report that N-type inactivation mediated by DPP6a and DPP10a is sensitive to redox regulation of Cys-13, a highly conserved N-terminal cysteine, similar to redox regulation of N-type inactivation of other Kv channels. Experiments using two oxidants (tBHP and diamide) yield similar functional effects and dose-response curves, in agreement with redox regulation. A difference develops between the tBHP and diamide effects at concentration above 250 μM, and it is most likely due to tBHP reacting with methionines as well as cysteines at higher concentration [43]. H_2_O_2_ has been shown to decreases peak current and accelerates P/C-type inactivation of ShakerB channels lacking N-type inactivation in part by oxidation of a methionine residue in the pore segment [54]. As for the reactive cysteine, interestingly it is found in DPP6a, DPP10a, and Kv1.4 at the same position 13, although in other proteins the reactive cysteine is more N-terminal than this (Fig. 1A and 1B). More experiments will be needed to determine whether the positions of redox-sensitive cysteines are critical to the molecular mechanism of oxidative regulation.

Our results from studies using *Xenopus* oocytes come with that caveat that these experiments were performed in a heterologous expression system using chemical oxidants. Further experiments will be needed to determine if a similar regulatory phenomenon is observed in native neurons using naturally occurring oxidation mechanism. We believe, however, that these results are likely to have general significance. First, Cys-13 is conserved in DPP6a and DPP10a subunits in species from fish to humans, suggesting there is an ancient regulatory mechanism (Fig. 1B). Second, it is clear that the intracellular milieus of *Xenopus* oocytes and mammalian cells have no significant impact on the redox chemistry of tBHP, diamide, or DTT. Redox studies using oocytes and two-electrode voltage clamp have repeatedly proven to be effective in examining the effects of cysteine oxidation on Kv channel gating; specifically, the redox regulation of Kv1.4 N-type inactivation has been demonstrated in both *Xenopus* oocytes and HEK-293 cells with the same effects [18], [55]. Third, the S-glutathionylation of Cys-13 of DPP6a or DPP10a is a reaction process that requires only the reactive cysteine and glutathione and thus likely takes place in either oocytes or mammalian cells.

### Oxidation of DPP6a and DPP10a Cys-13 by tBHP and diamide does not produce disulfide bridges

By analogy with Kv1.4, we began with the hypothesis that oxidative regulation of DPP6a-mediated N-type inactivation occurs by disulfide bridge formation. Using a biochemical approach, we found no direct evidence indicating that oxidation of DPP6a in the Kv4.2-KChIP3a-DPLP ternary complex by tBHP or diamide leads to a disulfide bridge between one DPP6a and a neighboring DPP6a or any other protein. Oxidized DPP6a does not exhibit an increase in apparent molecular weight as would be expected for formation of a disulfide bridge with nearby proteins. Indirect functional testing by co-expression and systematic mutational studies showed that the redox effects do not depend on cysteines on KChIP3a, Kv4.1, or Kv4.2, with the possible exception of Kv4.2 Cys-320. However, the C320A findings could not be replicated by mutation of the homologous residue C322 in Kv4.1 channels, and interpretations of the C320A results are complicated by the mutation producing significant effects on the inactivation, including an alteration in the voltage dependence of inactivation kinetics. The most likely explanation is that the effects of C320A are an artifact of a previously described acceleration of slower closed state inactivation process [56], which may accelerate slow inactivation to be nearly as fast as N-type inactivation and thereby masking any potential redox regulation of N-type inactivation.

Overall, our data indicate that oxidative regulation of DPP6a or DPP10a N-type inactivation, unlike the mechanism proposed for Kv1.4, is not due to protein-protein cysteine disulfide bridge formation. In agreement with our conclusion, published results show that Kv1.4 and DPP6a mediate N-type inactivation that responds differently to oxidizing conditions produced by patch excision. N-type inactivation of Kv1.4 channels is suppressed rapidly by patch excision and restored by patch cramming or by fast application of GSH [7]. In contrast, Kv4.2 ternary channel complex containing DPP10a inactivate with similar kinetics in either the cell-attached or inside-out configuration without GSH in the bath [12]. The DPP10a-mediated N-type inactivation remained intact in the excised patch configuration and permitted pharmacological testing of its mechanism.

### S-Glutathionylation of Cys-13: A new molecular mechanism of ROS-mediated regulation of N-type inactivation

An exciting finding in our current work is the discovery that oxidation by tBHP and diamide produces S-glutathionylation of Cys-13 in DPP6a and DPP10a N-termini. tBHP and diamide are two model compounds commonly used in studies to examine oxidative stress, and, in the natural cellular environment, it has been reported that H_2_O_2_, tBHP and diamide prefer to oxidize intracellular GSH and induce S-glutathionylation [44], [45], [51], [52], [53]. To prevent the formation of intracellular protein disulfide bonds and to detoxify oxidants, intracellular GSH is maintained at millimolar concentrations (5 to 10 mM) in the reduced form by a cytosolic NADPH-dependent reaction catalyzed by glutathione reductase [23]. Thus, H_2_O_2_, tBHP, and diamide would tend to first oxidize reduced GSH, increase GSSG levels, and dramatically increase the GSSG/GSH ratio. In addition, S-glutathionylation is a major reaction product of diamide treatment since diamide prefers to oxidize GSH thiols over protein thiols, because protein thiols are generally less acidic and more sterically hindered [44]. Finally, the similarity between the nominal EC50 values for tBHP and diamide suggests that they act via the same reaction pathway, perhaps with GSSG as the intracellular oxidizing agent. An important for future study is whether S-glutathionylation plays a central role *in vivo* in the redox regulation of A-currents. Although previous studies have suggested disulfide linkages are important for redox regulation of Kv1.4 and Kv3.4, S-glutathionylation was not a well appreciated mechanism when these earlier studies were performed.

As far we know, our work represents the first example of S-glutathionylation regulating Kv channel function, although reversible protein S-glutathionylation has been recently documented to regulate Na-K pump, IP_3_ receptor, cystic fibrosis transmembrane conductance regulator (CFTR) chloride channel, and KATP channel [37], [57], [,58], [59]. It is not completely clear how S-glutathionylation of Cys-13 produces the observed slowing of inactivation and increase in current, but it is highly conceivable that attachment of the GSH tripeptide disrupts the ability of the N-type inactivation ball domain to reach its pore binding site, due to either the size of the attached group or an electrostatic charge effect. Structural studies on Kv channels have shown that the N-terminus must enter the pore through relatively long and tortuous side window openings to reach the pore block inactivation [60], [61]. A tripeptide attachment to the N-terminus could easily affect the ability of the N-terminus to enter the side windows. Alternatively, the side window openings contain a concentration of negative charges. GSH contains a negatively charged glutamate at its N-terminus (GSH is in the globally monoanionic form GSH-, with positive amino group and two negative carboxylate groups), and this charge may impeded the entry of the blocking inactivation particle from passing through the channel side window openings. More experiments will be needed to test these hypotheses.

### Implications of oxidative modification for neuronal I_SA_

The published literature has established that, unlike Kv1.4, the endogenous N-terminus of Kv4.2 produces fast inactivation that is insensitive to redox [14], [15]. Consequently, redox-sensitive A-type currents in neurons is thought to be likely expressed by Kv1.4 rather than Kv4 channels [62]. Our heterologous expression studies strongly suggest that such interpretation should be reconsidered, since the Kv4-based subthreshold A-type current in neurons expressing DPP6a or DPP10a is likely also redox sensitive. Moreover, with a mixture of DPLPs being expressed in different neurons with different redox sensitivities, our results point to the importance of knowing the precise subunit composition in order to predict the functional and regulatory properties of the native current. Furthermore, since DPP6a-conferred N-type inactivation of Kv4 channels is regulated via S-glutathionylation, follow-up studies to examine redox regulation of I_SA_ in neurons or Kv4.2+KChIP3a+DPP6a currents in cultured mammalian cells must maintain normal intracellular GSH concentration to permit S-glutathionylation. This is an important consideration since, in the whole-cell patch configuration, the intracellular solutions dialyses out the intracellular milieu, and without GSH supplementation, there may be insufficient GSH for S-glutathionylation to produce normal regulation in such experiments.

Finally, our results emphasize the potential link between I_SA_, ROS, and oxidative stress-related disorders and the need for further studies. In neurons, ROS is robustly produced as a by-product of normal aerobic metabolism and carefully kept in check by antioxidants such as GSH. At physiological concentration, ROS contributes to the induction and long-term potentiation, synaptic plasticity, learning and memory, and normal cognitive functions [63]. Because I_SA_ is also a major contributor to many of the same neurological phenomena modulated by ROS, our results would suggest that ROS may perform its function through reversible oxidation-reduction of DPP6a and DPP10a I_SA_ auxiliary subunits. Under pathological conditions, an excessive accumulation of ROS results in neuronal oxidative stress, leading to apoptosis and reduced cognitive functions. An intriguing association between ROS and I_SA_ has been reported in cerebellar granule (CG) cells where DPP6a is highly expressed [13], [33]. Exposure of CG cells to low external potassium concentrations leads to increased ROS levels, decreased cell viability, increased I_SA_ amplitude, and slowing of inactivation kinetics [64], [65]. These effects are especially interesting since they are similar to those reported here as results of ROS oxidation of Kv4.2+KChIP3a+DPP6a channels. Since oxidative stress and apoptosis contributes to development of age-related neurodegenerative diseases such as Alzheimers disease, Parkinsons disease, Amyotrophic lateral sclerosis, and Hungtingtons disease [66], [67], further studies on how I_SA_ channels that contain DPP10a or DPP6a are regulated by protein oxidation may increase our understanding of their molecular pathology.
